# Pedagogical value and emotional impact of fitness testing in secondary physical education: student and teacher perspectives

**DOI:** 10.3389/fspor.2026.1701809

**Published:** 2026-02-16

**Authors:** Ryan T. Nolan, James F. Barkell, Louisa R. Peralta

**Affiliations:** The University of Sydney (Faculty of Arts and Social Science, Sydney School of Education and Social Work), Sydney, NSW, Australia

**Keywords:** fitness education, fitness testing, PE, physical education, secondary school, student voice

## Abstract

**Introduction:**

Fitness testing is widely used in school physical education (PE) yet is often criticised for demotivating students and being misaligned with educative aims. This study investigates its enactment in a “critical case”: a high socioeconomic status (SES), independent boys' school with extensive resources and a strong sporting culture. Theoretically, if the dominant performance-oriented testing model were to succeed anywhere, it should be in this “ideal” context.

**Methods:**

Semi-structured interviews with PE teachers (*n* = 7) and focus groups with Year 8 students (*n* = 11) were analysed using reflexive thematic analysis with Self-Determination Theory (SDT) as a sensitising lens.

**Results:**

Three themes emerged: (1) a Curriculum-to-Practice Disconnect, driven by a “hidden curriculum” of Talent Identification that displaced educative aims; (2) Emotional Dimensions, where public testing necessitated teacher “buffering” to mitigate shame; and (3) a Motivational Gap, where administrative pressures transformed teachers into auditors, providing data without actionable feedback.

**Discussion:**

The study demonstrates that “active demotivation” is a structural defect of fitness testing that persists even in resource-rich environments. We argue that adding resources without changing the pedagogical model merely shifts pressure from equipment scarcity to time scarcity. To address this, we propose a set of Refined Design Principles, mapping need-thwarting practices to evidence-based alternatives to support a shift from Performance-Oriented Testing to Process-Oriented Fitness Education.

## 1 Introduction

Fitness testing remains one of the most contested practices in school physical education (PE), described as “one of the most debated PE-for-health practices” (1) and “deeply entrenched” in pedagogical traditions across the UK, North America, and Australia (2). Critics argue that it privileges performative, individualised outcomes, risks demotivating students, and can undermine broader educational aims (3–5). While often defended for purposes such as accountability and health promotion (6), research indicates that teachers rarely use testing to build students' health-related knowledge or to foster lifelong activity habits (7). As Cale and Harris (4) suggest, it may ultimately be a misdirected strategy for promoting healthy lifestyles.

Yet experiences of fitness testing are far from uniform. Student responses are shaped by factors such as gender, fitness level, and socioeconomic background (8–10). Boys often report more positive attitudes than girls (8, 9), and higher-performing students tend to associate fitness testing with enjoyment and competence (11, 12). Because students from higher SES backgrounds generally achieve stronger fitness outcomes (10), those in high-SES boys' schools may perceive fitness testing more positively. This duality highlights a tension: while widely criticised, fitness testing may be affirming and motivating in contexts where students are perceived to be confident and competent.

In Australia, the official learning area is Health and Physical Education (HPE), which reframes PE around broader aims that include wellbeing, intrinsic motivation, critical inquiry, and lifelong movement engagement (13). At Year 8, fitness is named explicitly, for example: “participate in physical activities designed to improve fitness and wellbeing to investigate the impact of regular participation on health, fitness and wellbeing (AC9HP8M04)” and “design and justify strategies to increase physical activity levels to achieve health and wellbeing outcomes (AC9HP8M06)” (14). Although the curriculum does not prescribe “fitness testing” or name “fitness education,” these statements locate fitness within educative aims rather than as an isolated measurement task (13, 14). The curriculum also supports intrinsic motivation by promoting “dispositions that support lifelong physical activity participation” and “meaningful movement experiences” that foster self-directed engagement (13). Combining health and PE into a single learning area is a distinctive Australian and New Zealand approach (15). Here, “PE” refers to the movement and activity components, whereas “HPE” refers to the broader Australian curriculum that includes health education. In this study, “curriculum” refers to Australian HPE curriculum unless otherwise stated.

Quality PE is widely recognised as encompassing cognitive, affective, social, and physical outcomes (16), though this is not a global consensus. Definitions of quality PE vary internationally, and as Martins et al. (17) note, global monitoring and surveillance efforts often face a “misalignment of conceptual frameworks” and produce inconsistent data. The Australian HPE curriculum reflects this holistic vision, aligning with the UNESCO framework by including outcomes beyond psychomotor skills alone. In ACARA's HPE, this is set out through two strands (Personal, social and community health; Movement and physical activity) and key propositions that include Value Movement and Include a critical inquiry approach (13).

However, the extent to which these aims are realised depends on classroom enactment, and when enacted through fitness testing, tension arises between curricular intentions and practice. This tension reflects a long-noted policy–practice “slippage” in HPE, where external factors and habitual practices displace formal curriculum aims (18). This slippage and misalignment persist as teachers prioritise standardised, measurable outcomes (19) over the curriculum's intent for student inquiry and reflection (1).

At the level of classroom enactment, teachers acknowledge the shortcomings of fitness testing yet continue to use it in traditional formats (20). Despite recognising limited educational value, many reported using it because it is quick, standardised, and produces (perceived) measurable data that helps justify PE's place in the curriculum (2, 7). Teachers also admitted that fitness test results are rarely revisited or meaningfully used in their teaching, noting that data is sometimes discarded altogether (7). While these practices seem practical in busy school environments, they risk turning fitness testing into a tick-box exercise rather than a learning sequence guided by the curriculum with a clear educative purpose. These enactment patterns have clear implications for how students perceive and engage with testing. Students report unclear purposes, minimal explanation, little feedback, and limited integration with class learning, and often experience testing as judgement rather than support (12).

Students also have the capacity to discover new approaches to fitness testing that are responsive to contextual needs (21). This emphasis on student voice and agency is aligned with a holistic perspective, foregrounded in the concept of a “balanced approach” to PE (22). This approach positions fitness testing as a tool for teaching cognitive concepts, enhancing motor skills, and improving perceived competence through self-improvement and task mastery (22, 23). These principles are exemplified in a recent intervention study by Silva et al. (24) where a fitness education model was implemented with 12th-grade students, a demographic that has been observed to experience a decline in self-esteem and attitudes toward fitness testing (12). The study found that this student-centred approach, which included a full testing battery and emphasised self-planning, teamwork, and decision-making, significantly boosted student engagement and increased their fitness levels. Students were found to move from a state of dependence to one of greater autonomy, with the crafting of their own fitness plans emerging as a pivotal moment of engagement (24).

The emotional discomfort, motivational barriers, and pedagogical limitations identified in the literature also align with constructs from Self-Determination Theory (SDT) (25), a widely used framework that explains how social and environmental conditions shape students' motivation and wellbeing. According to SDT, meaningful engagement is more likely when three basic psychological needs are supported: autonomy (a sense of ownership and control over one's actions), competence (feeling capable and effective), and relatedness (feeling connected and supported by others). Vasconcellos et al. (26) demonstrated that satisfaction of these needs strongly predicts student motivation and engagement in school PE. However, while SDT has been widely applied in PE contexts, its specific application to fitness testing remains limited. Furthermore, evidence suggests that students have the capacity to discover new approaches to fitness testing that are responsive to their own needs when given the opportunity to collaboratively critique and reflect on current practices (21).

Curriculum alignment is widely recognised as crucial for effective PE, yet research identifies persistent discrepancies between educational objectives, instruction, and assessment (27). Drawing on SDT, this study examines how fitness testing practices may support or undermine students' motivation and engagement in PE. The research responds to a critical gap in existing literature, which has largely examined student and teacher experiences in isolation. By bringing these two perspectives together within the same school context (a high-SES boys' school), this study offers insights into the complex tensions that exist in a setting where positive experiences might be assumed. The findings aim to inform the development of design principles for fitness education.

Guided by these aims, we asked: (1) how students and PE teachers in a high-SES boys' school experience and make sense of fitness testing in PE; and (2) to what extent current fitness practices at this school align with the Australian HPE curriculum's educative intentions.

## 2 Methodology

This study adhered to the Standards for Reporting Qualitative Research (SRQR) checklist (28) to ensure transparency, rigour, and comprehensiveness in reporting. Ethical approval was obtained from the University of Sydney Human Research Ethics Committee (No. 2024/HE000184).

### 2.1 Research design

This study adopted a pragmatic approach, drawing on multiple theoretical and methodological frameworks to address the needs of the school context. Rather than committing to a single research paradigm, it prioritised practical usefulness by selecting concepts and methods for their relevance and value to the research problem. As Dudley (29) explains, such methodological flexibility reflects a pragmatic stance that favours doing what works over rigid adherence to a single way of thinking.

Specifically, the study was situated within an Educational Design Research (EDR) framework, which emphasises iterative cycles of design, implementation, analysis, and refinement (30). Building on a prior systematic review (31), this qualitative phase served as the diagnostic stage, exploring how students and teachers experience fitness education and testing in practice. These insights were used to inform the ongoing refinement of design principles to guide future fitness education programs in school PE.

In addition to EDR, the study drew on Practitioner Inquiry to support critical reflection on the taken-for-granted role of fitness testing in schools. Framed by Luguetti et al. (32) as inquiry as stance, this approach positions the educator not as a passive recipient of policy, but as an agentic researcher capable of challenging the “hidden curriculum” of performance-oriented testing, positioning students as active contributors to learning. These principles shaped the reflexive stance of the study, ensuring that both student and teacher voices were central to the research process. The researcher's insider position provided contextual insights, complemented by regular team-based reflexive discussions that critically questioned assumptions.

Finally, the study engaged with SDT (25) through a pragmatic and sensitising lens rather than a rigid deductive framework. In the design phase, SDT concepts informed the interview guide, prompting inquiry into students' sense of control (autonomy), their perceived capability and understanding of the “why” (competence), and their sense of belonging in PE (relatedness). During analysis, coding was primarily inductive, with SDT used as sensitising concepts to guide interpretation by asking: How might this reported experience be understood as supporting or thwarting a student's psychological needs? This allowed us to interpret student narratives of embarrassment or disengagement not just as emotional reactions, but as indicators of deeper motivational dynamics.

#### 2.1.1 Researcher reflexivity

The research team possessed extensive experience in school-based HPE teaching. The primary researcher (RN), an insider-practitioner with 16 years' experience, inevitably influenced all research stages. While facilitating open dialogue, this insider position posed a risk of influencing participant responses based on perceived researcher expectations. While providing rich contextual insight, this insider position may also have led to unconscious assumptions about the school's culture that were so familiar as to go unquestioned. The critical perspectives of external co-authors were therefore essential to challenge these assumptions and broaden interpretation.

Explicit reflexivity was practised, acknowledging positionality rather than attempting to eliminate it. RN's perspective, that fitness testing can be beneficial yet carries risks, guided balanced interview questioning. This view was informed not only by extensive teaching experience but also by prior empirical research on adolescent fitness declines during remote learning (33). Co-authors external to the research context contributed by challenging interpretations. One brought academic expertise in fitness education research, while another offered broad educational insights from diverse settings. Their involvement strengthened trustworthiness, surfacing otherwise unchecked assumptions.

We explicitly acknowledge the power dynamics inherent in practitioner research. To mitigate expectancy effects, where students might offer “correct” answers to please a teacher, data collection was deliberately scheduled at the very end of the academic year, after semester reports had been finalised and submitted. This temporal separation severed the link between participation and assessment. Furthermore, the teacher had cultivated a classroom climate of inquiry throughout the year, similar to Luguetti et al.'s (32) description of “inquiry as stance,” where students were accustomed to critiquing pedagogy without fear of reprisal. This established rapport arguably facilitated richer disclosure regarding emotional harm than an external researcher could have achieved.

#### 2.1.2 Setting and participants

The study was conducted in a single-sex, male independent school in Victoria, Australia. The site was selected via purposive sampling, based on the primary researcher's employment at the school, consistent with the Practitioner Inquiry (32) and Educational Design Research (EDR) (30) methodologies framing this study.

While selected for accessibility and contextual depth, the school theoretically functions as a “critical case” (34). Flyvbjerg describes a “most likely” critical case as one where “if this is (not) valid for this case, then it applies to all (no) cases” (34). With an Index of Community Socio-Educational Advantage (ICSEA) rating over 1,180, the school serves a student population from socio-educational backgrounds well above the national average (35). Furthermore, many staff hold coaching roles, students are highly familiar with competitive sport, and fitness testing results are formalised as letter grades in end-of-semester reports. These factors create what might be considered the “ideal conditions” for fitness testing to succeed (9, 10). Consequently, the study proceeds on the logic that if the negative experiences identified in existing literature are observed even in this “ideal” setting, it would demonstrate that favourable demographics and resources alone are insufficient to safeguard against the pedagogical risks of fitness testing.

##### 2.1.2.1 Student participants and sampling

We used purposive sampling to select participants on characteristics relevant to the study aims (36, 37). We defined the sample universe as Year 8 boys taught by one PE teacher and set inclusion criteria accordingly. We then approached students to ensure heterogeneity in fitness level and sporting background, allowing comparison across contrasting cases (36, 37). In addition, the first author (the class teacher) drew on his knowledge of students' communication styles and group dynamics to identify information-rich cases while avoiding over-dominant pairings, and we prioritised students who could participate respectfully without dominating discussion and who could understand and respond to questions (36, 37). We recognise that the teacher-researcher role may shape recruitment; to enhance rigour, sampling criteria were set in advance, and selections were reviewed within the research team, with decisions documented in a reflexive record.

Eleven parents/guardians provided consent and students assented; the eleven students took part in three focus groups (*n* = 2, 4, and 5).

##### 2.1.2.2 Teacher participants

The PE department comprised nine teachers, including the first author; excluding the first author, eight were eligible, of whom seven participated (88%). Participants represented a range of experience (10–30 years) and roles (classroom teaching, curriculum coordination, leadership of school sport programs, boarding house supervision, and pastoral care). Two non-PE staff were consulted informally to clarify school context; their input was not included in the analysed dataset.

##### 2.1.2.3 Confidentiality, identifiers, and citation conventions

To protect confidentiality in this single-site study, quotations are anonymised with role-coded identifiers and minimal detail. PE teachers are labelled PE-T01–PE-T07; students are S1–S11. Numbering indicates quoted participants only; not all interviewees are quoted. We do not attach role descriptors, job titles, exact years of experience, specific sports/teams, or combinations of attributes to any teacher identifier, to minimise deductive disclosure. We took a minimum-necessary context approach for student quotations (favouring self-described experiences over performance details). A re-identification key is stored separately and will not be shared. All teacher interviews occurred in person on 26 November 2024 (one paired interview). Student focus group interviews occurred between the 2–4 December 2024.

#### 2.1.3 Data collection

All semi-structured interviews with teachers and focus group interviews with students were conducted face-to-face on the school campus. Conducting interviews in person was practical, as all participants were based at the same school. This approach also aligned with safeguarding considerations, particularly for student focus groups.

All teacher interviews were individual, except for one paired interview with the Director and Assistant Director of Sport. Interview durations are summarised in Table 1.

**Table 1:** Interview details.

Participant Group	Format	Sample Size (n)	Duration (Range)	Average Duration
PE Teachers	Individual/Paired	7	12:48–36:53 min	≈ 24 mins
Students	Focus Groups	11	18:25–40:37 min	≈ 29 min

Interviews and focus groups were audio-recorded using Zoom Video Communications, Inc. software on an Apple MacBook. Audio-only recording was used to ensure comfort and followed ethical approval guidelines. The recordings were transcribed verbatim using Zoom's automated transcription feature and checked for accuracy. Teachers received no compensation. Students were given an ice block as a token of appreciation as their focus groups were conducted on hot school days.

#### 2.1.4 Interview guide

A semi-structured interview guide was developed to explore the perspectives of teachers and students on fitness education and testing. The guide included open-ended questions designed to elicit detailed responses, supported by prompts and probes to encourage deeper reflection. The interview guide was underpinned by SDT and designed to explore participants' experiences of autonomy, competence, and relatedness in the context of fitness testing and education. The guide was also informed by a prior systematic review (31) and shaped by the researcher's position as a teacher within the school. It was developed collaboratively and piloted with one teacher to ensure clarity and flow.

For teachers, the guide covered their practices, perceptions of fitness education and testing, communication with stakeholders. Teachers were also asked explicitly about how their practices supported students' basic psychological needs, with specific prompts regarding student choice (autonomy), feedback structures (competence), and the inclusivity of the learning environment (relatedness). Additional questions focused on the challenges faced in accommodating diverse needs, how fitness testing results were leveraged to inform teaching practices or curriculum adjustments and professional development in supporting teachers' implementation of autonomy-supportive practices.

For students, focus group interviews aimed to explore personal experiences and perceptions of fitness testing through the lens of SDT. Questions invited students to share their likes and dislikes, strategies for staying motivated, and memorable experiences—both positive and negative. The guide specifically addressed emotional responses such as discomfort or anxiety, investigating how teacher practices or peer interactions influenced their sense of safety and capability. Finally, students were invited to suggest improvements to enhance their learning experience and motivation.

#### 2.1.5 Data analysis

Reflexive Thematic Analysis (RTA) was employed following Braun and Clarke's (38) six-phase process, with the analysis also shaped by their later guidance that highlights the co-constructed nature of meaning and the researcher's active interpretive role (38). The process was primarily inductive, with themes developed from patterns generated, rather than imposed theoretical categories. However, SDT was used as a set of sensitising concepts; specifically, the research team interrogated the data by asking whether reported experiences (e.g., public testing) likely supported or thwarted the students' needs for autonomy, competence, and relatedness. The researcher's dual role as teacher and investigator provided contextual insight that was balanced through ongoing reflexivity and collaborative dialogue within the research team.

The primary data sources were interview and focus group transcripts. In Phase 1 (Familiarisation), the lead researcher (RN) immersed themselves in the data by reading and re-reading all transcripts multiple times, making initial notes on both expected and unanticipated ideas. Particular attention was given to how students and teachers described emotional responses, the perceived purpose of fitness testing, and the interpersonal dynamics surrounding it. In Phase 2 (Generating Initial Codes), RN manually coded the data in Excel. The coding was primarily inductive and aimed to retain participants' original phrasing where possible, using *in vivo* codes such as “it's a bit weird” *and “*just another stat you measure.” Although codes were developed from the data, they were interpreted through the lens of the research questions, relevant literature, and the sensitising concepts of SDT (particularly when participants referred to motivation, perceived capability, or social connection).

In Phase 3 (Searching for Themes), RN grouped related codes into broader conceptual clusters using spreadsheet-based categorisation and visual mapping. Initial clusters were tentative and descriptive, such as “emotions and testing,” “student motivation,” and “curriculum relevance,” and provided a foundation for identifying patterns across the dataset. These groupings were refined through multiple iterations, with RN revisiting coded extracts to assess their internal coherence and how well they captured the meanings expressed by participants. Reflexivity was sustained throughout, with deliberate consideration of how existing relationships and contextual familiarity could influence interpretation.

In Phase 4 (Reviewing Themes), developing themes were examined in consultation with co-researchers. This collaborative process provided a further layer of critical reflection, encouraging the refinement, consolidation, or removal of themes to enhance clarity and distinctiveness. In alignment with a “Big Q” approach to qualitative research (39), the analysis did not seek a single objective truth or consensus on coding accuracy. Instead, the research team engaged in a process of “collaborative reflexivity” over the course of three scheduled meetings. Co-authors challenged the primary researcher's interpretations to ensure themes were not merely topic summaries, but meaning-based interpretive stories. Disagreements were resolved not by voting, but by returning to the raw data to explore rival interpretations and develop a richer, more coherent narrative.

In Phase 5 (Defining and Naming Themes), RN worked collaboratively with the research team to finalise the definitions, names, and boundaries of each theme. These decisions were guided by the study's research questions, which focused on how students and teachers experienced and made sense of fitness testing within a school context. Themes were evaluated for their coherence, relevance, and ability to capture meaningful patterns across the data. Representative participant quotes were selected to illustrate the scope and nuance of each theme, ensuring the final thematic structure remained grounded in participants' voices.

In Phase 6 (Producing the Report), the refined themes were integrated into an interpretive narrative that responded directly to the research aims. While grounded in participants' voices, this narrative was also informed by relevant literature and sensitised by SDT, particularly in relation to motivation, capability, and social connection. The goal was to generate practical insights for educational practice.

Throughout all phases of analysis, RN engaged in ongoing reflexive dialogue with co-researchers, critically examining assumptions, refining interpretations, and enhancing the credibility and transparency of the findings (38).

## 3 Results

Thematic analysis identified three key themes from the data: (1) Curriculum Relevance and Integration; (2) Emotional and Social Dimensions of Fitness Testing; and (3) Motivation, Self-Improvement, and Ownership. For each theme, we examine both student and teacher perspectives, highlighting points of alignment and divergence and integrating practical implementation barriers and solutions. Consistent with the study's analytic approach, we interpret these themes in relation to the HPE curriculum's educative intent, specifically, the requirements to participate in activities designed to improve fitness and wellbeing (AC9HP8M04) and designing and justifying strategies to increase physical activity (AC9HP8M06), as well as the propositions of value movement and critical inquiry (13, 14).

**Figure 1:**
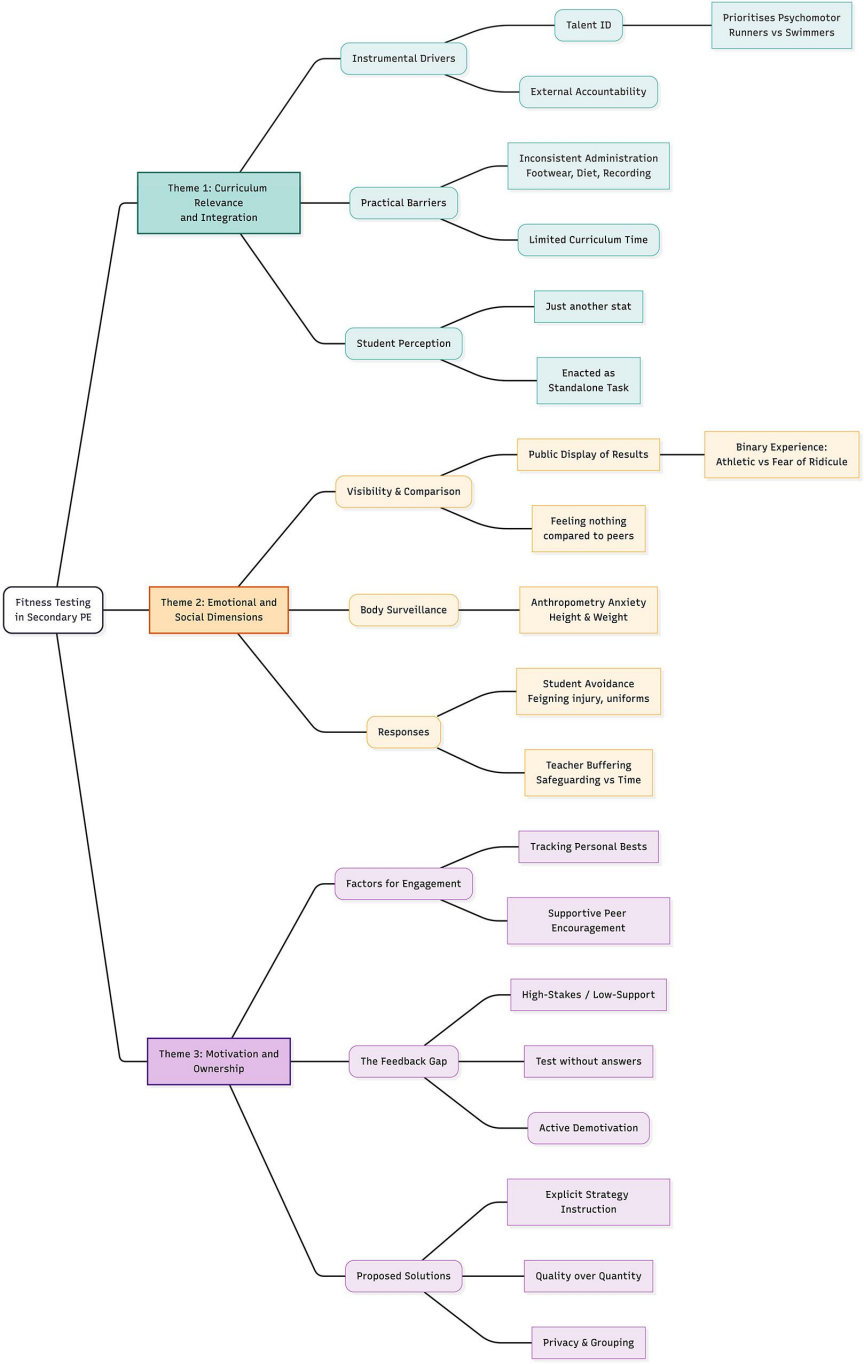
A thematic map illustrating the key analytical dimensions of fitness testing in secondary PE. The map categorises findings into three distinct themes. Read from top to bottom, the arrangement mirrors the study's narrative arc: systemic drivers (Theme 1) set the conditions for the emotional (Theme 2) and motivational (Theme 3) experiences enacted in the classroom. Left-to-right directional lines indicate the hierarchical breakdown of specific practices and student perceptions within each theme. Colour coding differentiates the dimensions: Green represents Curriculum Relevance (Theme 1); Orange represents Emotional and Social Dimensions (Theme 2); and Purple represents Motivation and Ownership (Theme 3).

To illustrate the structural relationships between these findings, Figure 1 presents a thematic map of the analysis. This figure visualises the intricate interweaving of the data, mapping how instrumental drivers (Theme 1) precipitate specific affective burdens (Theme 2) and motivational constraints (Theme 3), alongside the practical barriers that reinforce these outcomes.

### 3.1 Theme one: curriculum relevance and integration

This theme captures perceptions regarding fitness testing's educational relevance, highlighting a critical gap between current practices and desired educational outcomes. The persistence of this isolated approach appears driven by instrumental pressures from stakeholders such as parents and school leadership, rather than by genuine pedagogical intent.

Students consistently perceived fitness tests as disconnected from broader educational aims, unclear in purpose, and lacking meaningful context. Student S1 critically described fitness testing as something that “just feels like another stat you measure,” underscoring the perception of testing as externally driven rather than genuinely educational. Student S2 questioned the educational value of specific tests, stating, “Sometimes it's hard to see the point of certain tests, like the basketball throw… we don't know how it measures our overall fitness or why it matters”. Similarly student S3 felt the tests were without purpose, noting the disconnect between action and understanding: “We have no idea of what we're doing. we know we're doing it, but we don't know why”.

Teachers corroborated these concerns, acknowledging that fitness testing was rarely revisited or systematically integrated into broader HPE curriculum activities. For example, PE-T04 admitted tests were not effectively connected back into regular PE content, stating “apart from reporting, I don't really refer to them (fitness test results) at all.” PE-T02 noted that administrative constraints meant previous attempts to provide detailed profiles had been “axed” due to lack of support, similarly adding “we do the fitness tests at the start of the year, then jump back into usual sports. We don't always tie back to, hey, remember your beep test results”. This highlights a curriculum-level tension where testing functions as a standalone measurement activity.

Further reinforcing this instrumental orientation, teachers identified talent identification, a predominantly psychomotor outcome, as the primary driver for any integration that did occur. PE-T07 summarised this dynamic, noting that the program “stands almost in isolation, with the exception of the link between athletics, where it is used for talent ID”. PE-T05 explained that tests were specifically selected for: “targeting boys to different sports areas” and “push[ing] them into sports. based on their body shape and size” noting “athletics, swimming, and power-oriented sports such as rugby union as areas of focus.” However, this focus on selecting specific athletic traits created perceived inequities, rewarding a narrow skill set while alienating others. PE-T07 acknowledged that the testing battery disproportionately privileged land-based athletes, explicitly admitting that “*our* acknowledgement of a swimmer is far less than a boy who gets 16 on the beep test”. This hierarchy of value was acutely felt by students; Student S9 argued that despite being a high-performing swimmer, “you look at [the report].. it's not fair” that their domain of expertise is invisible to the testing battery. Similarly, Student S8 noted that comparisons felt “unfair” when testing against peers who matured earlier, resigning himself to the fact that “you're never going to be able to beat them, because they're bigger than you”. Consequently, the curriculum risks narrowing from broad education to a mechanism for sorting bodies based on key psychomotor outcomes, validating only those whose physical attributes serve the school's specific talent identification goals while marginalising others.

Teachers also identified significant practical barriers, notably inconsistent testing administration. When asked about challenges of fitness testing PE-T06 raised reliability issues, answering “ consistency of results across different people administering the tests in different ways”. PE-T01 similarly raised reliability concerns, describing practical administration challenges: “we've got boys sometimes doing beep tests in school shoes or bare feet.. or just after lunch where they've had a really big meal.” He further explained the difficulty of large-group testing: “Trying to keep track of.. 20 boys doing the test correctly at the same time is really tricky, and.. can be subject to error.” PE-T02 agreed, noting discrepancies where “you saw them do 7.1 and they've written down 8.1”. Such inconsistencies can confuse stakeholders and undermine pedagogical value and measurement reliability, limiting meaningful comparisons and educational insights. When asked for solutions PE-T07 simply recognised a lack of consistency as “the ongoing challenge” and others suggested adopting digital data systems to improve administration and support curriculum integration.

In summary, the data reveals a stark misalignment between practice and curriculum. Testing is enacted as a standalone measurement task rather than a learning sequence for fitness and wellbeing (AC9HP8M04), resulting in students consistently reporting a lack of understanding regarding its purpose. These gaps are reinforced by an instrumental focus on talent identification, which privileges a narrow set of physical attributes over broad educational value, alienating students whose capabilities lie outside the school's target sports. However, a critical contradiction emerges here: while teachers utilise these results to “target” and select students for specific sports, they simultaneously acknowledge that the data is compromised by significant reliability issues. Addressing this inconsistency is essential to realise the educational potential of fitness testing.

### 3.2 Theme Two: emotional and social dimensions of fitness testing

This theme explores participants' accounts that fitness testing, particularly when public or highly visible (as they are commonly enacted), can sit at odds with PE’s wellbeing aims by eliciting embarrassment, self-consciousness, and fear of judgement.

Testing frequently provoked feelings of exposure, with the visibility of results creating a stark divide between students. Student S4 articulated this vulnerability: “It's a bit weird with everyone really finding out your fitness score. It feels embarrassing if people see you’re getting off [the beep test] really early”. Student S5 explained that this public display creates a binary experience: that competition could motivate effort but simultaneously cause stress “if I'm a really athletic kid, then I'm gonna be fine with it, but if I'm not so athletic… you're afraid they might make fun of you”. This discomfort extended beyond performance to body surveillance; Student S11 recounted a specific instance regarding height measurement: “I personally found it really embarrassing.. because this year I had to go after [a taller peer].. I found it pretty embarrassing”. Student S6 explained that the public display of results amplified this vulnerability, noting that public announcements can “make them feel nothing compared to those people.. [and] make people feel uneasy”. This discomfort was a recurring sentiment among students, suggesting that harm is not incidental; it can emerge when testing is public, comparative, or graded, and may reflect broader structural issues within PE.

Teachers corroborated these accounts, observing that students often employed avoidance strategies to manage this anxiety. PE-T01 noted a consistent pattern of disengagement among older students, listing specific tactics: “ They've got an injury that's just popped up.. they won't bring their uniform.. they'll say they've got a cough”. He described the prevailing student sentiment as “a sigh rather than an excitement”, identifying “fear of failure, but also fear of judgment” as the root causes. Teachers recognised the need to frame testing positively but cited practical constraints, particularly limited time for individualised support. As PE-T06 put it, while “some love it,” others ask “why are we doing this?”—yet critically, “we don't have time to sit down and talk about that in a meaningful way”. PE-T03 echoed this pressure, noting that often “it's just a case of doing the tests,” and by the time they are finished, “it's time for the boys to go and get changed”. This highlights the immense pressure teachers face.

Critically, these discussions reveal a deeper underlying issue: fitness testing seems uniquely problematic among school activities, requiring continual, deliberate effort by teachers to manage or “buffer” potential emotional harm. PE-T06 emphasised the need for strict protocols during anthropometric testing: “I'll never, ever ask them to read out heights, weights.. no opportunity.. for them to be singled out or ridiculed”. Similarly, PE-T03 noted the difficulty for students “knowing that.. there's a lot of students in the class that will notice.. they're not that fit”. This practice suggests that unlike typical educational tasks, fitness testing inherently risks student discomfort or humiliation, fundamentally misaligning with contemporary educational values prioritising wellbeing and inclusivity.

Students proposed practical safeguards to mitigate these risks. Student S5 suggested allowing anxious students to test “at a lunch time.. where no one else sees them,” while Student S10 proposed “putting people in groups of like fitness level.. so [you] wouldn't be too upset if they just destroy you”. Student S4 added regarding body measurements: “it would be best for some people that would like to keep it private”. These suggestions imply that while small adjustments help, a more fundamental reconsideration of testing practices may be needed to prioritise student safety and dignity.

In summary, participants' accounts point to misalignment with wellbeing-oriented curricular intentions: when testing is public, comparative, or graded, it risks discomfort that sits at odds with the HPE content statements emphasising meaningful participation and supportive learning environments. Furthermore, a clear gap remains between intent and impact: while teachers acknowledge these vulnerabilities and attempt to “buffer” harm through protocols, students continue to report significant distress and avoidance, compelling them to propose their own structural solutions.

### 3.3 Theme three: motivation, self-improvement, and ownership

Here, we use ownership to mean students' felt responsibility for goals and progress, which we distinguish from SDT's autonomy (a broader sense of volition and choice). This theme addresses perceptions of fitness testing's role in fostering intrinsic motivation, self-improvement, and ownership, and the conditions under which testing contributes to (or undermines) educational goals such as personal growth and lifelong physical activity engagement. This connects to the misalignment identified in Theme One: when curricular purpose is unclear, motivation and agency are undermined.

Participants identified goal setting and tracking personal bests as central to fostering intrinsic motivation and ownership. Students particularly valued repeat testing to track their progress. Student S5 explained, “If you do one at the beginning of the year and one at the end, you can see if there's improvement you weren't aware of”. Two PE teachers (PE-T06; PE-T01) agreed that seeing gains can be an “impetus for improvement”. However, students were highly critical when testing occurred without subsequent learning. Student S3 offered a powerful critique of this feedback gap, stating: “That is like doing a test.. and just giving a test back with a score and no marks.. if we do the fitness test and we go, alright, this is what you can do to improve.. That'd be massively helpful”. This analogy highlights a fundamental pedagogical flaw: students view current testing protocols as assessment of learning (a final score) rather than assessment for learning (a tool for growth).

Some students distinguished stressful competitive comparisons from supportive peer encouragement. Student S3 described how encouragement among peers during testing helped motivation: “Alright, how are we going? We're exhausted. Let's just do another one”. These insights suggest peer interactions during testing can be beneficial when framed as supportive rather than comparative. Conversely, Student S11 rejected empty praise, noting that “pity points just don't help anybody,” preferring honest comparison to drive improvement.

Teachers emphasised that assigning scores alone did not sustain motivation, highlighting the importance of ongoing goal setting and personalised feedback. PE-T02 warned that simply awarding a grade at year's end without feedback can “damage [confidence] and deter them away from physical activity”. This reflects a common pedagogical tension: while teachers aim to support behaviour change, practices often remain narrowly focused on psychomotor outcomes (e.g., better scores). As PE-T01 admitted, “We just do the tests and that's the score, and they’ll see their score on the report, and we just require on their own… internal feedback to interpret that result and sort of make improvements from their own perspective.” PE-T07 embodied this tension between ideal and reality: while he asserted that “you actually need to give steps to take.. [so they] know what to do to get fit”, he simultaneously admitted that regarding goal setting to impact behaviour, he had “never done that”. This contradiction reveals that the failure to foster ownership is not due to a lack of teacher knowledge, but a lack of pedagogical structures to support it.

Teachers identified practical barriers that entrenched this lack of feedback (and therefore undermined motivational aims), including limited PE time and rising administrative demands. PE-T04 noted, “time is our biggest challenge.. we don't have the luxury of being able to spend significant amount of time with the boys in that space”. Consequently, students are left without the tools to act on their results. As PE-T05 noted, “If we do a beep test once, but never revisit how they can improve… some kids might feel deflated”. By conducting testing as a purely administrative task, providing the “diagnosis” of a grade without the “prescription” of how to improve, the current approach risks confirming students' perceived lack of competence rather than building their capacity. Student S3's plea for the “answers to the test” reveals a hunger for ownership that is currently being thwarted by logistical constraints and curricular misalignment.

To bridge this gap, participants proposed practical solutions to restore educational value. Student S3 suggested that explicitly teaching “strategies to do them” would reduce anxiety and improve performance, while PE-T04 proposed a structural trade-off: “making some positive change.. by maybe doing less tests.. but doing ones that are a bit more meaningful”. These suggestions acknowledge that logistical constraints diminish fitness testing's motivational and educational potential. Consequently, stakeholders appear willing to sacrifice data volume for data depth, prioritising the quality of the educational experience over the quantity of metrics collected

In summary, participants recognised that fitness testing can foster motivation, support self-improvement, and build ownership only when embedded in structures that emphasise personal goals, supportive interactions, and actionable feedback. Yet, as enacted, the practice often operates inversely: prioritising data collection over student development. Stronger curricular alignment, clear learning intentions, opportunities to plan and justify activity strategies, iterative participation, and reflective inquiry would better realise the intent of the HPE curriculum. This would enable motivation, self-improvement, and ownership to be sustained over time while building movement competence and cognitive understanding.

## 4 Discussion

The findings of this study reveal a significant and complex misalignment between the intended educative purposes of PE and the practical enactment of fitness testing in a high-SES, single-sex male school. Drawing on a top-down, policy-to-practice narrative, our discussion synthesises three central themes: (1) Curriculum Relevance and Integration, which shows a persistent curriculum disconnect driven by instrumental pressures and compounded by inconsistent administration; (2) Emotional and Social Dimensions of Fitness Testing, where public and comparative enactments expose students to embarrassment and social risk, necessitating constant teacher “buffering”; and (3) Motivation, Self-Improvement, and Ownership, which are strengthened by goal setting, feedback, and supportive interactions but are constrained by time and performance-first practices. Consistent with our analytic approach, we interpret these findings through the lens of SDT, specifically drawing on the classification of Teacher Motivational Behaviours (TMBs) established by Ahmadi et al. (40) to theorise the specific mechanisms of need-support and need-thwarting observed.

While students and teachers recognised potential educational value in fitness testing, this value tended to emerge only when enactment was participatory, purposeful, and protective of wellbeing. Conversely, current practices often functioned as “need-thwarting” events. Taken together, the data suggests that fitness testing is too often a standalone measurement task that privileges psychomotor outcomes over the curriculum's propositions of value movement and critical inquiry (13).

We discuss each theme in turn, drawing out implications for curricular alignment and practical enactment in this context.

### 4.1 The curriculum-to-practice disconnect

The central finding of this study is a persistent disconnect between the educative purpose of the curriculum and the instrumental enactment of fitness testing. In practice, testing was frequently experienced by students and described by teachers as a task-oriented, standalone event. This perception is consistent with Alfrey's (21) finding that students often struggled to identify what they were meant to learn from fitness testing and echoes her call for clearer explanations of its relevance within PE. Consequently, the practice creates a performative focus: students engage in the act of testing without engaging in the learning of fitness. This narrow implementation aligns with Alfrey and Landi's (1) observation that fitness testing remains one of the most contested PE-for-health practices, in part because it is frequently delivered in isolation with minimal attention to its educative value or connection to broader learning outcomes.

A critical driver of this disconnect in the current context was the prioritisation of Talent Identification (Talent ID). Teachers described selecting fitness tests to identify athletic potential to direct students toward particular sports, often based on body shape, size, or explosive power (PE-T05), reflecting a focus on talent pathways rather than broader educative aims. We theorise this prioritisation as a competing “hidden curriculum” that functions as a form of need-thwarting teaching behaviour. Drawing on the classification system by Ahmadi et al. (40), the practice of evaluating students based on genetic attributes aligns with the behaviour of criticising a fixed quality (Code CT2). By focusing on innate traits rather than effort, and by using data to stream students into ability groups (Grouping students on the basis of ability, Code CT9, the hidden curriculum teaches students that fitness is a fixed trait to be measured, rather than a skill to be developed. This creates an environment that actively thwarts the need for competence among those who do not fit the athletic ideal (e.g., swimmers in a running-based battery).

This emphasis on talent identification reflects what Landi, Walton-Fisette, and Sutherland (41) describe as a dominant “orientation” within PE, one shaped by performance and accountability pressures that crowd out learning activities that are tailored to develop students' affective, cognitive, and social learning capabilities. As demonstrated in international contexts, accountability-driven testing has been shown to narrow pedagogical focus and reduce student engagement (19).

This disconnect is further compounded by “intra-departmental variability,” where rationales for testing differed markedly even within the same faculty. This mirrors international evidence that consensus on the “why” and “how” of fitness testing is often weaker than its prevalence suggests among teachers, who often acknowledge limits to the educative value of testing even as they continue to use it (7, 20). Taken together, these patterns point to a teacher-centred, test-led model that makes the educative purpose opaque for students, a problem repeatedly identified in recent work (21). Moving toward a more student-centred approach will likely require structural support and a shared vision within the department, not just individual teacher intent (42).

Finally, these inconsistencies call into question the educational validity of the practice. Teachers raised concerns about unreliable testing conditions (e.g., varied student attire, insufficient time) which compromise the data's legitimacy. This is a crucial point, as without consistent protocols, even genuine improvements may go unrecognised, undermining the very reason students expressed they valued the tests. As Johnson et al. (43) and Templeton and Korchagin (10) argue, when data collection takes precedence over pedagogical purpose, the opportunity for meaningful, individualised learning is lost. While reliable student-led testing is possible with training (44), the present findings suggest that even among experienced teachers in a high-SES, well-resourced school, without structured reflection and deliberate integration, fitness testing risks becoming a procedural exercise lacking authentic value (24).

### 4.2 Emotional harm and the imperative to “buffer”

The disconnect between curricular intent and enactment creates significant emotional labour for teachers, who described a need to “buffer” students against the potential harm of public testing. This mirrors findings from previous research documenting negative emotional responses to fitness testing (1, 8, 12). While this 'shame' is well-documented, our data highlights how this is exacerbated in the current context by a masculine performance culture where public failure is equated with social vulnerability.

The “beep test” remains the focal point of this anxiety. Prior work likewise identifies the beep test as especially problematic for student affect (9), and the avoidance behaviours observed in our study (e.g., feigning injury) echo longstanding concerns raised by Hopple and Graham (45), suggesting such patterns persist. We interpret this persistence through the lens of Ahmadi et al. (40), identifying the beep test's elimination format as a form of competence thwarting (Code CT1), as it involves publicly presenting critical feedback. Ahmadi et al. (40) note that such public visibility increases the risk of feedback becoming ego-threatening.

However, the harm extended beyond the beep test to the standardised nature of the fitness testing battery itself. Because the testing protocols apply the same metrics to all boys regardless of maturation or body type, they function as an undifferentiated challenge (Code CT5), where the same task is assigned to all students regardless of their ability level. This lack of differentiation inevitably highlights biological disparities, functioning as a critique of a fixed quality (Code CT2), a behaviour Ahmadi et al. (40) describe as emphasising innate or genetic traits required for success rather than effort. This was acutely felt by Student S11 regarding anthropometric measurements, who found it embarrassing because he “had to go after [a taller peer]”. Similarly, Student S6 noted that public announcements of these results made students “feel nothing compared to those people.”

In our study, students and teachers independently raised similar concerns, but their proposed changes diverged. Teachers described “buffering” strategies intended to reduce harm, including careful language, individual rather than public feedback, and reduced visibility of results, while also noting limited time to address anxieties (PE-T03; PE-T06). Interestingly, students proposed structural changes that diverge from standard SDT recommendations. Student S10 explicitly requested “putting people in groups of like fitness level” to avoid the shame of being “destroyed” by superior peers. Theoretically, this request for Grouping students on the basis of ability (Code CT9) contradicts the consensus of the Ahmadi et al. (40) Delphi panel, which generally classified such grouping as need-thwarting because it publicly signals low competence. However, Ahmadi et al. (40) acknowledged that experts disagreed on this specific behaviour, noting that ability grouping can sometimes facilitate appropriate differentiation.

In the context of a high-performance boys’ school, students appear to view ability grouping not as a marker of shame, but as a protective mechanism against the greater threat of undifferentiated public comparison (CT5). This aligns with Safron and Landi's (46) argument that students often experience fitness testing as a site of intense “public judgement” and surveillance. In such a hostile climate, the theoretical downside of segregation is outweighed by the immediate need for safety. Thus, ability grouping becomes a pragmatic survival strategy to mitigate the structural risk of “public judgement” illustrating a tension between ideal pedagogical theory and the lived reality of the testing environment.

These findings highlight a pedagogical paradox: current testing practices are so structurally threatening that students actively practice avoidance strategies and request segregation as a harm-reduction strategy. While teachers attempt to “buffer” these harms individually, this reactive approach contrasts with the proactive shift to a student-centred model advocated by Alfrey (21). Such a model would prioritise student wellbeing by design rather than relying on ad-hoc harm reduction. However, implementing this shift requires teachers to take on additional responsibilities to mitigate harms within a practice that remains embedded in programs, raising questions about its ongoing justification. Crucially, the need for constant buffering strategies underscores a central contradiction: fitness testing, promoted as an educative practice, requires ongoing harm-reduction measures to avoid negative consequences. This suggests that a more fundamental reconsideration of testing practices may be needed to prioritise safety and dignity.

### 4.3 The motivational and pedagogical Gap

In this school, four positions highlight a conceptual tension around fitness testing: (1) teachers' desire for behavioural change; (2) their reliance on performance-based measures; (3) students' preference for supportive, autonomy-oriented environments; and (4) the absence of systematic follow-up. The disconnect between these positions creates a chain of cause and effect in which performance-driven assessment undermines motivation, despite intentions to foster wellbeing and long-term behavioural change.

Our findings advance the literature by characterising this disconnect not merely as ineffective, but as actively demotivating. Students described a feedback gap where they received a score but no guidance on improvement, a sentiment captured perfectly by Student S3's analogy of “doing a test and not telling you the answers.” From an SDT perspective, this practice represents a failure to support competence. Effective feedback should be Competence Supportive (Code CS2), defined by Ahmadi et al. (40) as providing feedback targeting specific improvement strategies. By providing a “diagnosis” (the score) without the “prescription” (the strategy), the current practice risks confirming a student's lack of ability without offering a pathway to change it.

This failure to provide the “answers to the test” extends to a lack of resources for independent growth. Teachers acknowledged that while they hoped testing would change behaviour, they rarely provided the necessary scaffolding, such as providing extra resources for independent learning (Code AS11) or teaching students to set intrinsic life goals (Code AS7). Consequently, testing becomes the intervention rather than a pedagogical starting point, despite evidence that durable change depends on interpretation, goal setting, and structured practice (2, 7, 24).

In the absence of teacher-led guidance, students often looked to each other for scaffolding. While some interactions were negative, Student S3 noted that supportive peer encouragement helped them persist through exhaustion (“Let's just do another one”). This aligns with Safron and Landi (46), who argue that while testing environments can be high-risk, supportive peer dynamics can mitigate negative emotional responses. However, without deliberate pedagogical structuring, these positive interactions are left to chance rather than design.

Crucially, this feedback gap appears driven by structural barriers rather than a lack of teacher knowledge. Teachers admitted they “don't have time” for goal setting (PE-T07), a reality that transforms the testing event into a high-stakes audit. This aligns with Alfrey and O'Connor (42), who discuss how resourcing and structural constraints hinder pedagogical change, and Harte et al. (7), who found that teachers struggle to align testing with educational goals in the face of such pressures.

The impact of these barriers was embodied by PE-T07, who articulated a sharp contradiction between ideal and reality: while he asserted that “you actually need to give steps to take.. [so they] know what to do to get fit”, he simultaneously admitted that he had “never done that”. This contradiction reveals that the failure to foster ownership is systemic. As noted by Student S11, “pity points” (empty praise) do not help; students desire honest, strategic comparison. Without the pedagogical structures to support specific feedback (CS2) and praise for improvement (CS3), fitness testing risks becoming a confirmation of fixed incompetence.

Finally, recognising the interplay between this lack of feedback and the emotional safety concerns highlighted in Section 4.2 helps explain why both groups emphasised the importance of student-centred approaches. As Silva et al. (24) highlight, fitness testing only becomes educationally meaningful when students are actively engaged in interpreting their results. Without this engagement, the potential for motivation is lost to the structural reality of the feedback gap.

### 4.4 Summary and implications

These findings show that the motivational potential of fitness testing is unlikely to be realised without the deliberate integration of goal setting, progress tracking, and personalised feedback into the HPE curriculum. Ensuring these elements are embedded in practice is essential for fostering student ownership, sustained engagement, and meaningful learning outcomes.

Consistent with an EDR framework, these diagnostic findings serve as the foundation for refining design principles for future enactment. While recent systematic reviews (31) have identified broad pedagogical principles, such as the need for Meaningful Assessment and Intentional Instruction, the current study reveals the specific contextual barriers (such as the feedback gap and public shaming) that prevent these principles from being realised in practice.

Therefore, we propose a set of refined design principles to guide future implementation. Table 2 operationalises the broad theoretical aims of fitness education into specific, actionable protocols. By mapping the need-thwarting practices observed in this study to specific need-supportive alternatives drawn from Ahmadi et al. (40), this matrix transforms pedagogical goals into precise shifts, such as moving from public critical feedback to private progress tracking.

**Table 2:** Refined design principles [adapted from ahmadi et al. (40)]: mapping current practices to teacher motivational behaviours, with proposed implementation checkpoints and success indicators.

Current Practice (Observed Barrier)	SDT Consequence	Refined Design Principle & Implementation Checkpoint	Pedagogical Benefit & Success Indicator
Public beep test: Students run until exhaustion in front of peers.	Competence Thwarting: Criticising/comparing via peer comparison (CT1, CT3).	Private Support & Empathy (CS10, RS1, RS6): Test in private/small groups; Show unconditional positive regard (RS1) and understanding of student feelings (RS6). Checkpoint: Adoption of non-public protocols with flexible options (e.g., small groups, partner recording, or self-paced stations) to remove the “audience effect” while maintaining validity.	Meaningful Assessment: Mitigates shame/social risk; prioritises safety over surveillance. Success Indicator: A measurable decrease in observable avoidance behaviours (e.g., absenteeism, “forgotten” uniforms, or feigned injuries) specifically on testing days.
Talent ID Selection: Testing used to slot boys into sports based on body shape.	Competence Thwarting: Criticising/Emphasising a fixed quality (CT2).	Self-Referenced Goals (CS7, CS3): Set goals based on self-referenced standards (CS7); Praise improvement or effort (CS3). Checkpoint: Introduction of a “Fitness Strengths Inventory” where students map their attributes to activities they might suit, shifting focus from recruitment to personal discovery.	Theoretically Grounded: Shifts focus from genetic sorting (fixed mindset) to personal growth. Students can identify a personal physical strength and a corresponding activity they enjoy, regardless of their “A-team” status.
The Feedback Gap: Providing a score/grade with no follow-up strategies.	Competence Thwarting: Absence of feedback aimed at improvement (Omission of CS4).	Feedback for Improvement (CS4, CS14): Provide feedback aimed at improvement (CS4); Facilitate self-monitoring of progress (CS14). Checkpoint: Dedicated “Data Interpretation” time where students map their scores to “Healthy Fitness Zones” (Criterion-referenced) rather than just peer rankings (Normative).	Intentional Instruction: Ensures students have the “answers to the test” (strategies). Success Indicator: Students can verbally explain what their score indicates about their health status (e.g., “I am in the healthy zone,” not just “I beat him/her”).
Unexplained Protocols: Students unsure of the test's purpose or relevance.	Autonomy Thwarting: Absence of rationale (Omission of AS3).	Rationale & Expectations (AS3, CS11): Provide explanatory rationales (AS3); Clarify expectations of the learning task (CS11). Checkpoint: Explicit instruction mapping each test to its specific Fitness Component (e.g., “We do this to check Cardiorespiratory Endurance”).	Curricular Integration: Links testing to broader health concepts; reduces confusion. Success Indicator: Students understand how fitness tests measure certain fitness components.
Standardised Battery: All students do the same test regardless of relevance.	Autonomy Thwarting: Activities that exclude some students (AT2).	Allow for student input or choice (AS1) in test selection. Checkpoint: Implementation of a “comparative battery” where students attempt multiple valid protocols (e.g., both Handgrip and Flexed Arm Hang) to explore different expressions of strength.	Autonomy Support: Moves from compliance to needs-supportive teaching. Success Indicator: Students experience competence by identifying tests where they excel, while simultaneously learning that some tests inherently favour specific physiques (reducing feelings of failure).
No Resources for Growth: Testing without scaffolding for independent improvement.	Autonomy Thwarting: Failure to support independent learning (Omission of AS11).	Resources for Self-Monitoring (AS11, CS14): Provide resources (AS11) to support self-monitoring of progress and effort (CS14). Checkpoint: Provision of longitudinal logbooks (digital or paper) used across multiple terms/years.	Measurement Literacy: Helps students reason from “what I did” to “what changed.” Success Indicator: Reflections shift from outcome-focused (“What did I score?”) to process-focused (“How did I improve?”).

While this study argues that pedagogical reform does not strictly require financial resources, we acknowledge that the transferability of these principles to resource-constrained contexts requires adaptation. As Templeton and Korchagin (10) highlight, disparities in funding and infrastructure are systemic barriers that disproportionately affect schools serving economically disadvantaged communities. For example, regarding facilities, teachers can utilise station-based circuits where fitness testing is one of several concurrent activities to effectively diffuse the 'spotlight effect.’ Additionally, regarding equipment costs, teachers can implement comparative batteries by selecting from the wide range of valid, equipment-free protocols (e.g., bodyweight measures) that do not require expensive tools. Similarly, while well-resourced contexts may utilise digital tracking for feedback, low-resource contexts can achieve the same ownership outcomes through simple paper-based logbooks. The critical factor is not the technology or the facility, but the pedagogical shift from surveillance to support.

While these principles address immediate pedagogical reform, broader questions remain. Future research should examine how fitness testing experiences vary across diverse school contexts, including different SES levels, co-educational settings, and varied cultural backgrounds to test the transferability of these findings. Participatory and co-design approaches that actively involve both teachers and students could help create more motivationally supportive and contextually relevant fitness testing practices (32).

Additionally, system-level enablers, such as clear policy frameworks, adequate resourcing, and targeted professional development, warrant further investigation to determine how they can best support the curricular integration of fitness education. Furthermore, longitudinal research is needed to assess whether the specific SDT-aligned reforms proposed in Table 2 lead to sustained improvements in students' intrinsic motivation, engagement, and long-term physical activity participation.

## 5 Limitations

Several limitations frame the interpretability of these findings. First, the exclusive focus on male students means the findings may not capture how gender influences experiences of fitness testing (8, 9). Second, the relatively small sample size constrains the breadth of perspectives represented, potentially limiting the detection of more nuanced or divergent experiences. Third, although the researcher's insider position was addressed through explicit reflexivity, it may still have influenced participants' responses. Finally, the study remained exploratory and did not assess the effectiveness of any implemented reforms.

A significant contextual boundary is the study's location in a high-SES, independent boys' school. We theorise that the prioritisation of Talent ID observed here is structurally linked to the school's strategic drive to maximise sporting success to reinforce its elite brand. This aligns with Greaves et al. (47), who suggest that in competitive markets, schools often leverage visible extracurricular achievements to distinguish themselves from rivals. Consequently, the active demotivation observed here may be distinct to hyper-competitive, resource-rich environments where physical competence is a currency of identity. While low-SES contexts may struggle with resource scarcity (10), our findings suggest that high-SES contexts struggle with conflicting interests, where abundant resources are directed toward performance outcomes rather than inclusive education.

## 6 Conclusion

This study examined fitness testing in what could be considered a critical case: a high-SES boys' school with extensive resources, a strong sporting culture and a motivated student body. Theoretically, if the dominant performance-oriented testing model were to succeed anywhere, it should have been here. The fact that need-thwarting practices (40), emotional distress, and disengagement were prevalent even in this “ideal” setting provides a powerful indictment of the practice itself. It suggests that the failures of fitness testing are not merely symptoms of resource scarcity or student apathy, but are structural defects in the testing model that persist across socioeconomic divides.

While the outcomes of shame, anxiety, and disengagement mirror those found in diverse and low-SES contexts (10), our findings reveal that the drivers in this context are distinct. In this resource-rich environment, the “thwarting” stems from a conflict of purpose. The “hidden curriculum” of talent identification, driven by the school's strategic need to maintain its sporting brand (47), creates a hyper-competitive environment where the educative value of PE is crowded out by the instrumental need to sort and rank bodies.

Furthermore, this context exposes a critical paradox regarding teacher capacity. Despite having access to advanced facilities and technology, teachers remained “too busy” to provide meaningful feedback, a form of ambivalence and pedagogical 'slippage' often observed in fitness testing literature (20). The administrative demands of the “arms race” for data and performance transformed teachers into auditors, widening the feedback gap where students receive diagnoses without prescriptions. This confirms that adding resources without changing the pedagogical model does not solve the problem; it merely shifts the pressure from equipment scarcity to time scarcity (42).

Ultimately, this study contributes to the field by demonstrating that active demotivation is a systemic risk of performance-oriented testing, regardless of a school's privilege. Therefore, reform does not require more resources, but a fundamental repurposing of the test itself. By adopting the SDT-aligned reform model proposed in this paper, which shifts from performance-oriented testing to process-oriented fitness education, schools can ensure that fitness testing ceases to be a mechanism for sorting boys and becomes a meaningful vehicle for teaching them how to value, understand, and improve their health.

## Data Availability

The datasets presented in this article are not readily available because the materials are not shareable under our ethics approval. Requests to access the datasets should be directed to Ryan Nolan, rnol1294@uni.sydney.edu.au.

## Supplementary material

The Supplementary Material for this article can be found online at: https://www.frontiersin.org/articles/10.3389/fspor.2026. 1701809/full#supplementary-material
