# Supplementary material for: Pedagogical value and emotional impact of fitness testing in secondary physical education: student and teacher perspectives
Source: Front Sports Act Living. 2026 Feb 16;8:1701809. doi: 10.3389/fspor.2026.1701809 (PMC12950691; doi:10.3389/fspor.2026.1701809)
Supplement: Supplementary file 1 [file Supplementaryfile1.pdf]

Appendix A: Practitioner’s Guide

Transforming Fitness Testing into Fitness Education

The Reality Check: What our Students Told Us

We would never give a Maths test, read the marks out loud to the class, and then never teach the students how to fix their errors. Yet, our students told us that is exactly how fitness testing feels. As one student described it: *"It is like doing a test and not being told the answers."*

The Core Principle: Testing is for Learning

We need to treat fitness like any other subject. A test result shouldn't just be a score to record; it should be information that helps the student learn what to do next. If a student doesn't know *how* to improve their score, the test hasn't worked.

Five immediate changes to move from Testing to Education.

Instead of this... (The Old Way)	Try this... (The Education Way)	Why? (The Science)
**The Spectacle:**<br>Running the Beep Test as a whole class where everyone watches students drop out one by one.	**The Circuit:**<br>Run the test in small groups or as a "station" in a circuit. Use partner recording so only one peer sees the score.	**Lowers "Social Threat":**<br>Reduces the fear of public failure, which is the #1 cause of students feigning injury or forgetting uniforms.
**The "Blind" Score:**<br>Giving a student a result (e.g., "Level 6") with no follow-up.	**The Interpretation Lesson:**<br>Dedicate the lesson *after* testing to "reading" the data. Have students map their score to a health zone, not a peer rank.	**Builds Competence:** A score without a strategy is just judgment. Students need the "answers to the test" (how to improve).
**The "Cookie Cutter":**<br>Every student must do the exact same test (e.g., Pull-ups), even if their body type makes it impossible.	**The "Comparative Battery":**<br>Let students try two valid options (e.g., Handgrip vs. Flexed Arm Hang) and choose which one best captures their strength.	**Supports Autonomy:**<br>Teaches students that tests have biases (physics). Helps them find a "win" rather than confirming they are "bad at PE."
**The "Talent Scout":**<br>Using tests primarily to find the best athletes for school teams.	**The "Personal Inventory":**<br>Use tests to help students discover their *own* strengths. (e.g., "I'm not a runner, but I have great power for swimming").	**Shifts the "Why":**<br>Moves the goal from *institutional benefit* (winning trophies) to *student benefit* (lifelong health).
**The One-Off:**<br>Testing once a year for a report card.	**The Logbook:**<br>Use a simple paper or digital logbook where students record "Personal Bests" over time.	**Focuses on Growth:**<br>Shifts the mindset from "Who did I beat?" to "Did I beat my last score?"
